# Potassium Channels Kv1.3 and Kir2.1 But Not Kv1.5 Contribute to BV2 Cell Line and Primary Microglial Migration

**DOI:** 10.3390/ijms22042081

**Published:** 2021-02-19

**Authors:** Ruxandra Anton, Mihail Ghenghea, Violeta Ristoiu, Christophe Gattlen, Marc-Rene Suter, Petre Alexandru Cojocaru, Aurel Popa-Wagner, Bogdan Catalin, Alexandru-Florian Deftu

**Affiliations:** 1 Department of Anatomy, Animal Physiology and Biophysics, Faculty of Biology, University of Bucharest, 050095 București, Romania; antonruxandraelena@yahoo.com (R.A.); m.ghenghea@yahoo.com (M.G.); v_ristoiu@yahoo.com (V.R.); 2 Pain Center, Department of Anesthesiology, Lausanne University Hospital (CHUV) and Faculty of Biology and Medicine (FBM), University of Lausanne (UNIL), 1011 Lausanne, Switzerland; christophe.gattlen@gmail.com (C.G.); Marc.Suter@chuv.ch (M.-R.S.); 3 Department of Physiology, University of Medicine and Pharmacy of Craiova, 200349 Craiova, Romania; cojo.alexandru92@gmail.com; 4 Experimental Research Center for Normal and Pathological Aging, University of Medicine and Pharmacy of Craiova, 200349 Craiova, Romania; aurel.popa-wagner@geriatricshealthyageing.com; 5 Earth, Environmental and Life Sciences Division, The Research Institute of the University of Bucharest (ICUB), 050107 București, Romania

**Keywords:** microglial cells, potassium channels, migration, spared nerve injury, pain

## Abstract

(1) Background: As membrane channels contribute to different cell functions, understanding the underlying mechanisms becomes extremely important. A large number of neuronal channels have been investigated, however, less studied are the channels expressed in the glia population, particularly in microglia. In the present study, we focused on the function of the Kv1.3, Kv1.5 and Kir2.1 potassium channels expressed in both BV2 cells and primary microglia cultures, which may impact the cellular migration process. (2) Methods: Using an immunocytochemical approach, we were able to show the presence of the investigated channels in BV2 microglial cells, record their currents using a patch clamp and their role in cell migration using the scratch assay. The migration of the primary microglial cells in culture was assessed using cell culture inserts. (3) Results: By blocking each potassium channel, we showed that Kv1.3 and Kir2.1 but not Kv1.5 are essential for BV2 cell migration. Further, primary microglial cultures were obtained from a line of transgenic CX3CR1-eGFP mice that express fluorescent labeled microglia. The mice were subjected to a spared nerve injury model of pain and we found that microglia motility in an 8 µm insert was reduced 2 days after spared nerve injury (SNI) compared with sham conditions. Additional investigations showed a further impact on cell motility by specifically blocking Kv1.3 and Kir2.1 but not Kv1.5; (4) Conclusions: Our study highlights the importance of the Kv1.3 and Kir2.1 but not Kv1.5 potassium channels on microglia migration both in BV2 and primary cell cultures.

## 1. Introduction

Alongside astrocytes and oligodendrocytes, microglia represent the main glia population in the central nervous system (CNS). As the resident immune population of the CNS, microglia are constantly surveying their environment, permanently changing each individual arborization by generating new processes and retracting existing ones [1,2]. This also implies that microglia morphology can vary from region to region, with spinal microglia having a distinct morphology when compared to the microglia in the cortex [3]. In contrast, regardless of anatomical distribution, resting microglia bodies are relatively immobile in physiological conditions. Full body enlargement and migration usually occurs only as a response to injury [4]. Therefore, body migration is almost synonymous with microglia activation that will, in turn, initiate a long-lasting change in its physiology [5,6,7]. This change is beneficial during the acute phase of disease but could be detrimental on the outcome [8,9]. As such, balancing the timing, duration and intensity of the microglia response to damage is therapeutically relevant.

Many attempts have been made to modulate microglia inflammatory behavior [10]. However, due to the high number of signaling pathways that can potentially activate microglia [11], most of the experimental approaches have been only partially successful. The extent of microglia reactions and cellular mechanisms involved in certain pathologies such as stroke [12] and traumatic brain injury [13,14] are not clear. Further, although their involvement is more than apparent, the mechanisms still elude, as is the case with demyelination in multiple sclerosis [15] or the formation of amyloid beta deposits in Alzheimer’s disease [16,17]. In other pathologies, such as chronic pain after peripheral nerve injury, microglia activation can be observed [18,19] and seems to play a central role in pain persistence [20]. However, is not clear if activated microglia generates chronic pain or microglia activation is a consequence of pain. This uncertainty is because microglial cells produce chemokines and cytokines, and express their receptors as well, both being essential for cell migration [21], adhesion control and feedback to their own pro/anti-inflammatory state [22]. Furthermore, microglia produce a large amount of reactive oxygen species (ROS) and nitric oxide (NO) under conditions such as Aβ-induced oxidative stress and inflammation which may alter their migration rate [23], while the treatment with the natural compound eupatilin reduces BV2 microglial migration and the production of inflammatory cytokines and ROS species [24].

Understanding the basic roles of microglia receptors and ion channels can reveal important physiological and pathological functions of these cells, as well as for pain processing. For example, the purinergic receptors [25] have been associated with different functions including migration [26], phagocytosis [27] and cellular death [28]. Others, such as neurohormones and neuromodulators, including histamine receptors [29], opioid [30], neurotrophin [31], glucocorticoid and mineralocorticoid receptors [32] have no definite function in microglia physiology and were less investigated.

Experiments performed on microglia after facial nerve injury showed the regulation of potassium channels in the mechanisms underlying pain. One of the most important findings was the discovery of an inward rectifying potassium current mediated by Kir2.1, at 12 h after facial nerve axotomy [33]. Based on this discovery, it was proposed that Kir2.1 electrophysiology could be used as a potential marker of microglia activation [33,34]. This observation has led to other investigations that revealed, for example, the role of Kv1.5 in nitric oxide release by microglia after lipopolysaccharide (LPS) administration [35]. The Kv1.3 channel was linked to macrophage proliferation [36], cytokine release [37] and migration [38]. However, no experimental work has been carried out to confirm potassium channel involvement in microglia migration.

In the present study, the potassium channels Kv1.3, Kv1.5 and Kir2.1 were shown to be functionally present in the membrane of BV2 microglial cells, were labeled by immunocytochemistry and their current amplitudes were recorded using the whole-cell patch clamp approach. Moreover, Kv1.3 and Kir2.1 are implicated in the migration of these cells after the scratch wound healing assay. Primary microglial cells isolated from the ipsilateral spinal cord dorsal horn (SC-DH) after the spared nerve injury (SNI) model of pain migrated less than in sham conditions and their migration rate was impaired after blocking the investigated potassium channels. 

## 2. Results

### 2.1. The Characterization of Potassium Channels Kv1.3, Kv1.5 and Kir 2.1 in the BV2 Microglial Cell Line

**Figure 1:**
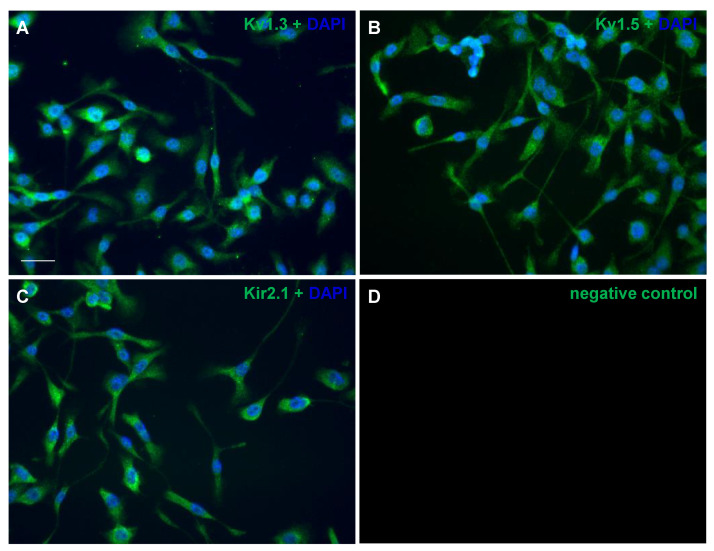
Immunolabeling of potassium channels in cultured BV2 microglial cells. The cells were stained with antibodies targeting Kv1.3 (**A**), Kv1.5 (**B**), Kir2.1 (**C**) and the negative control with Alexa 568 (**D**). The coverslips were mounted using the Prolong antifade with DAPI in blue. The channel was pseudo-colored in green for improved visualization. The images are representative of three independent experiments. Scale bar: 40 µm.

**Figure 2:**
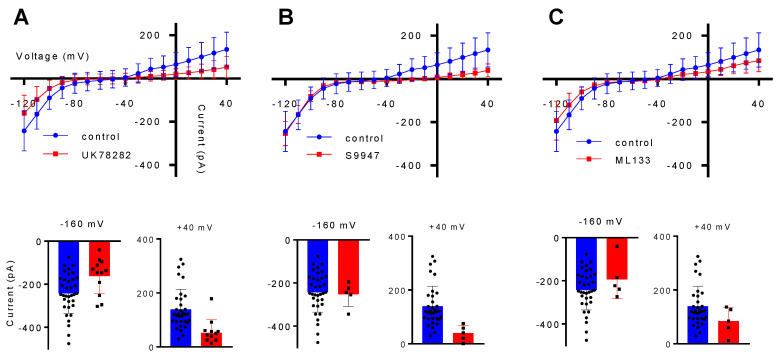
Electrophysiological properties of BV2 cells. For all Kv1.3, Kv1.5 and Kir2.1 potassium channels, the electrophysiological characteristics are represented by the intensity/voltage (I/V) curves extracted from the step recordings, representing the total current elicited by a voltage protocol starting from −120 mV, in 10 mV increments, to +40 mV (**A**–**C**), in control conditions and after each specific inhibitor. The inhibition effect of the of each blocker can be seen from the I/V curves. The insets represent the comparison of the current amplitude elicited by a voltage step at −160 mV and +40 mV. All the data are represented as Mean ± SD (*n* = 3 independent experiments).

As only a partial characterization of Kv1.3, Kv1.5 and Kir2.1 in the BV2 cell line was available, we confirmed both by immunocytochemistry and electrophysiology the presence of these channels in the BV2 cell line. The immunostaining analysis revealed that all of the investigated channels are expressed in the membrane of BV2 cells cultured on thin glass coverslips (Figure 1). However, the mere labeling of a cellular structure does not necessarily prove its function. As such, an electrophysiological characterization was made using a whole-cell patch clamp configuration. The intensity/voltage (I/V) curves were build based on the step protocol by quantifying the current dependent on the voltage, which ranged from −120 mV to +40 mV, with a 10 mV increment and 500 ms duration. Therefore, acute application for 3 min of 1 µM UK78282 for Kv1.3, 1 µM of S9947 for Kv1.5 or 2 µM of ML133 for Kir2.1 revealed that the BV2 microglial cell line functionally expresses the investigated potassium channels (Figure 2).

### 2.2. Potassium Channels Kv1.3 and Kir2.1 But Not Kv.1.5 Are Needed for BV2 Migration

**Figure 3:**
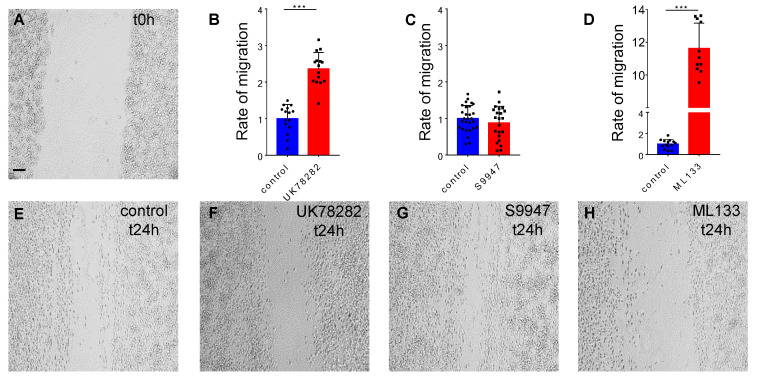
The contribution of potassium channels in BV2 microglial migration. The representative images show the robust and repetitive scratch made with the 200 µL sterile pipette tip at t0h (**A**) and the cell migration in control conditions (**E**) and after the inhibitor at t24h (**F**–**H**). The histograms show that BV2 microglial cells migrate less after the inhibition of Kv1.3 and Kir2.1 (**B**,**D**), whereas blocking Kv1.5 has no effect on cellular migration (**C**). Scale bar: 100 µm. All the statistical analysis is represented as Mean ± SD, ***: *p* < 0.001, using the parametric t-test (*n* = 3 independent experiments).

To investigate the impact of the Kv1.3, Kv1.5 and Kir2.1 potassium channels on the migration potential of BV2 cells, we used the scratch wound healing assay (Figure 3A). The control conditions at t24h were normalized to 1.00 ± 0.386, *n* = 15 (Figure 3B). Our experiments show that at t24h after the inhibition with 10 µM of UK78282, the free area of the scratch remained clearly increased to 2.36 ± 0.447, *n* = 15, *p* < 0.001 (Figure 3B,F) compared to control conditions, suggesting that blocking Kv1.3 in BV2 cells inhibits migration in this experimental setup. Blocking Kir2.1 with 20 µM of ML133 revealed the highest inhibition of migration, with a free area of 11.62 ± 1.542, *n* = 11, compared with controls 1 ± 0.424, *n* = 14, *p* < 0.001 (Figure 3D,H), indicating that this potassium channel might play a central role in microglial migration. Kv1.5 inhibition using 10 µM of S9947 did not show a difference in the migration rate under these experimental conditions, with a mean free area of 0.87 ± 0.457, *n* = 22, compared to control 1 ± 0.357, *n* = 28, *p* > 0.05 (Figure 3C,G).

**Figure 4:**
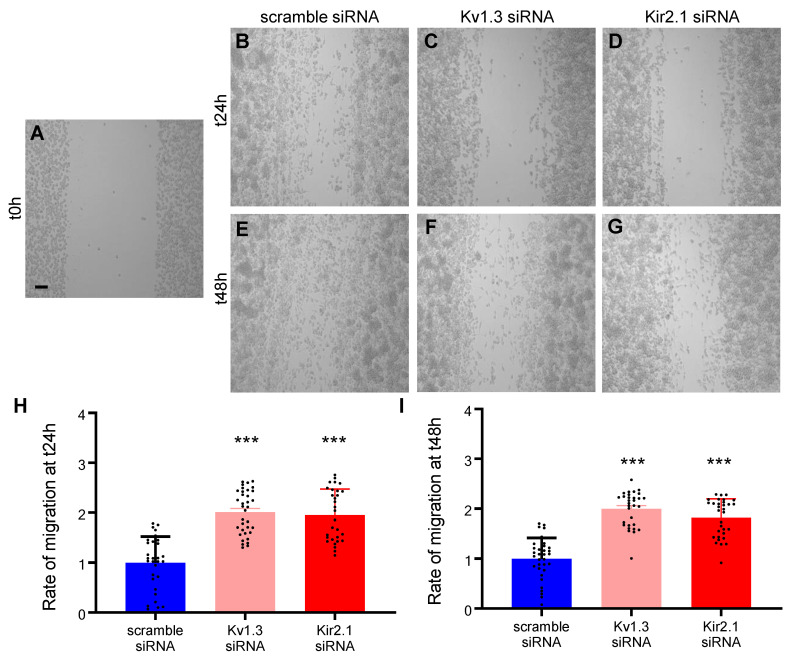
BV2 microglial migration after the silencing of the Kv1.3 and Kir2.1 channels. The representative images show the scratch made with the 200 µL sterile pipette tip at t0h (**A**) and the cell migration after scrambled and silencing siRNA at t24h (**B**–**D**) and at t48h (**E**–**G**). The bar graphs show the BV2 migration after scrambled and silencing siRNA at t24h (**H**) and at t48h (**I**). Scale bar: 100 µm. All the statistical analysis are represented as Mean ± SD, ***: *p* < 0.001, using the parametric t-test (*n* = 3 independent experiments).

Moreover, using the same scratch assay, we tested the contribution of only Kv1.3 and Kir2.1, the most potent channels linked to BV2 migration, after the transfection of specific silencing or scrambled small interfering RNA (siRNA). Our result suggests that at t24h after transfection, the rate of migration decreases. The free area of the scratch increased from 1.000 *±* 0.524, *n* = 31 when transfecting the scrambled siRNA (Figure 4A,B,H), at 2.009 *±* 0.437, *n* = 35, *p* < 0.001 after Kv1.3 siRNA (Figure 4C,H) and 1.953 *±* 0.52, *n* = 30, *p* < 0.001 after Kir2.1 siRNA (Figure 4D,H). The free area after the scratch remained still significantly high also at t48h after the transfection of scrambled siRNA 1.000 ± 0.4154, *n* = 33 (Figure 4E,I) compared with Kv1.3 siRNA 1.997 ± 0.3490, *n* = 31, *p* < 0.001 (Figure 4F,I) and Kir2.1 siRNA 1.821 ± 0.3768, *n* = 32, *p* < 0.001 (Figure 4G,I).

### 2.3. Microglia Have an Innate Decreased Motility after the Spared Nerve Injury

**Figure 5:**
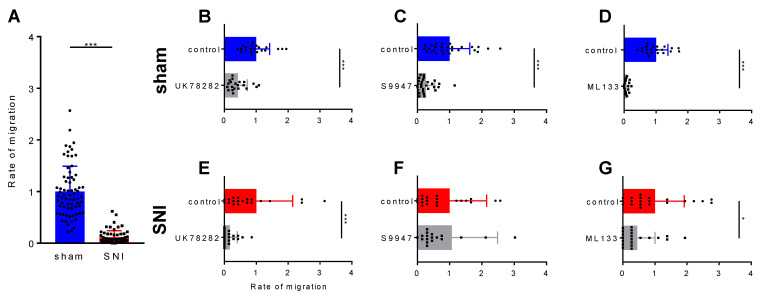
The migration of primary microglial cells through inserts with 8 µm pores. (**A**) The migration rate of primary microglial cells after the spared nerve injury (SNI) surgery is reduced compared with sham conditions. Histograms showing the contribution of each potassium channel, Kv1.3, Kv1.5 and Kir2.1, to microglial migration, in both sham (**B**–**D**) and SNI conditions (**E**–**G**). All the statistical analysis is represented as Mean ± SD, *: *p* < 0.05, ***: *p* < 0.001, using the nonparametric Mann–Whitney test (*n* = 3 independent experiments).

Next, we asked how the SNI surgery impacts microglia motility. To test this, we obtained primary microglial cultures from CX3CR1-eGFP transgenic mice [35] that were previously subjected to either a SNI or a sham surgery. Microglia were harvested from the ipsilateral SC-DH 2 days after surgery and cultured on inserts, in normal medium or with the inhibitor, for 3 h at 37 °C. All the experiments showed a decreased microglia motility after SNI, compared to the sham (*p* < 0.001) (Figure 5A). After establishing the importance of Kv1.3 and Kir2.1 in the migration rate of the BV2 microglial cell line in culture, we asked how the inhibition of these channels impacts the migration after the SNI surgery. As such, we used the specific inhibitors to target potassium channels and as expected, blocking Kv1.3 (Figure 5B,E) and Kir2.1 (Figure 5D,G) decreased microglia migration in both the SNI and sham conditions. Surprisingly, we found that the rate of microglial migration when blocking Kv1.5 was not changed in the SNI but still decreased in the sham conditions (Figure 5C,D). These results indicate that 2 days after the SNI surgery, microglial motility seems to be impaired and the inhibition of potassium channels decreased their migration even more, as shown in the complete analysis from Table 1.

## 3. Discussion

Although a number of studies have reported the presence of Kv1.3, Kv1.5 and Kir2 potassium channels in the membrane of microglial cells, their implication on cell migration has not been, to our knowledge, evaluated in detail. Therefore, the present study focused on investigating the impact of the three potassium channels on cellular migration in both the BV2 cell line and primary microglial cultures after the SNI model of pain. By using immunocytochemistry, we were able to (i) detect the expression of these potassium channels in cultured BV2 cells, (ii) show that Kv1.3 and Kir2.1 but not Kv1.5 are essential for BV2 cell migration by specifically blocking each potassium channel, (iii) record the electrophysiological properties of these channels in BV2 cells and (iv) to demonstrate their impact on microglia motility in primary cultures derived from a mouse model of neuropathic pain.

**Figure 6:**
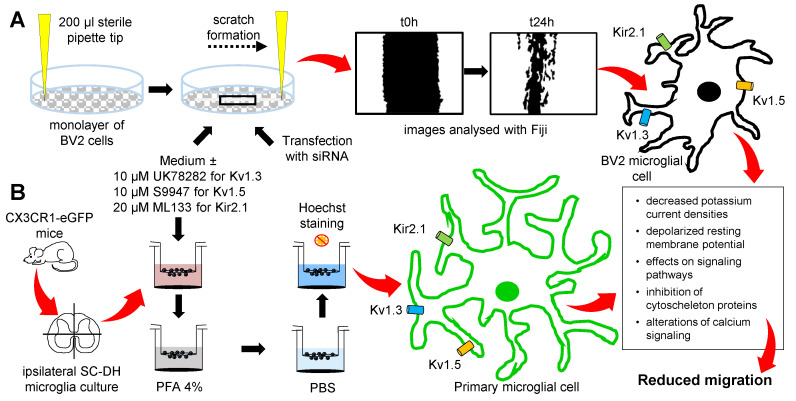
The experimental steps showing the inhibitory effect of potassium channels on microglial migration. (**A**) The monolayer of BV2 microglial cells was scratched with a sterile pipette tip and incubated with the medium or the pharmacological inhibitors or the cells were transfected with small interfering RNA (siRNA); pictures were analyzed at t24h and the inhibition of Kv1.3 and Kir2.1 reduced the rate of migration in the BV2 microglial cell line. (**B**) CX3CR1-eGFP transgenic mice were subjected to SNI or sham surgeries, the ipsilateral spinal cord dorsal horn (SC-DH) was dissected and cultured for 3 h in 8 µm pore inserts, in the culture medium or in the presence of the inhibitors, and the rate of migration was quantified in each condition. Different mechanisms by which the inhibition of the investigated potassium channels may influence the microglial migration rate, are proposed in the outlined box. PBS: phosphate buffer saline; PFA: paraformaldehyde.

In BV2 cells, Kv1.3 was shown to have an increased expression at 24 h after in vitro stimulation with 100 ng/mL LPS [39], while Kv1.5 was linked to microglial NO release and a decreased proliferation after LPS treatment in primary microglial cell cultures [38]. However, information on Kir2.1 function is scarce and the most common function observed for this channel is setting the resting membrane potential [40]. In our experiments, the expression and the function of these potassium channels were investigated in the BV2 microglial cell line. Thus, the low currents recorded in these cells indicate a small expression of the potassium channels at the plasma membrane, but still the inhibitory effect of the blockers can be seen and appreciated. Therefore, no statistical analysis was made, and the histograms are just a representation of the cell currents at the indicated voltage steps. Our study indicates that Kv1.3 and Kir2.1 change the BV2 microglial migration in the scratch assay, by changing the general currents in these cells or by changing the resting membrane potential to more depolarized values. These changes in the currents can translate to changes in the intracellular signaling pathways and the alteration of cytoskeleton protein (Figure 6) such as Iba1, an important actin crosslinking protein [41] or alterations in calcium signaling [42].

It is well known that every passage can change gene expression in cell line cultures, however, studies have shown that changes at the transcriptome level are less likely to happen in primary cell cultures [43]. Therefore, after showing that potassium channels contribute to BV2 migration, we were interested in confirming our results in primary microglia cultures. The choice to additionally use primary microglia cultures has also been motivated by reports showing differences between BV2 cells and primary microglia cultures [44,45]. Primary microglial cells express functional Kv1.3, Kv1.5 and Kir2.1 but their role may depend on their distribution in the CNS [11]. Although the endogenous expression of the investigated potassium channels is low in spinal cord microglial cells, nonetheless, there are reports showing their impact on the overall microglia activity [46,47,48]. Thus, investigating potassium channels in spinal microglial cells is important for our full understanding of microglia roles in the CNS. In this study, we have shown that microglia migration is modulated in the spared nerve injury and could be further impacted by the Kv1.3, Kv1.5 and Kir2.1 potassium channels. Thus, inhibiting Kv1.3, Kv1.5 and Kir2.1 in primary microglial cultures led to a decreased migration rate, further suggesting a contribution of these potassium channels to microglia motility. Interestingly, after SNI, although microglia are less mobile, inhibiting Kv1.5 does not further impact the microglial migration, most probably due to other unknown intracellular mechanisms. Hippocampal investigations have shown that in a proliferative environment, Kv1.5 channels can switch to endogenous Kv1.3 [36]. However, we were not able to find any report confirming these findings in the spine after SNI, but the lack of effect after blocking Kv1.5 could be explained by a similar change in the potassium channel expression.

Another limitation of the present study is the selectivity and specificity of the drugs used to inhibit the potassium channels. Although these molecules are reported to have a high affinity for the targeted channels and to have low IC50 values, we may encounter different actions on other ion channels, as ML133 was seen to have a small effect on Kir4.2 and Kir7.1 [49], while UK78282 can also disrupt Kv1.4 [50] and S9947 may also inhibit the Kv4.3 [51]. Therefore, the inhibition of other ion channels is not completely ruled out. To overcome this caveat, our experiments using the small interfering RNA technology, by which scrambled or silencing siRNA were introduced in BV2 cells, show that specifically knocking down the Kv1.3 and Kir2.1 reduced cellular migration.

Still, the most puzzling finding is that SC-DH microglial cells migrate less after the SNI surgery. Studies show that in vivo microglial cells migrate faster towards a laser-induced injury or ATP acute application and therefore suggesting that activated microglia survey the CNS and actively migrate to a specific injury/stimulus [1,52]. Our findings may be explained through the fact that SNI is a surgery in the peripheral nervous system and thus neuronal activation triggered upon cutting the nerves is increasing the neurotransmitter release at the level of the ipsilateral SC-DH and this release is arresting microglia in that region of activation. Thus, microglia may show an activation pattern resembling local activated cells that closely regulate the synaptic transmission in the SC-DH, but, of course, further experiments are needed to prove this hypothesis.

## 4. Materials and Methods

### 4.1. Animals and Surgery

All animal experiments were done under the EU Directive 2010/63/EU for laboratory research following the ethical guidelines of the University of Bucharest. Adult transgenic mice (>25 g) expressing the CX3CR1-eGFP membrane receptor [53] were used in these experiments. The mice were subjected to either a SNI or sham surgery under general isoflurane anesthesia. Briefly, following an incision through the biceps femoris muscle, the sciatic trifurcation was exposed. For sham conditions, a small 3 mm silk string was placed near the common peroneal and tibial nerves, while for SNI conditions the string was passed under these nerves, tightly ligated and the nerves were cut distal to the node and a 2 mm piece of nerve was removed. The sural nerve remained intact (spared) and untouched throughout the entire procedure. The muscle and the skin were separately sutured with individual nodes to avoid the opening of the wound by the mouse, who may scratch and bite the string, as previously described [54].

### 4.2. Cell cultures

#### 4.2.1. BV2 Cell Line Culture and Transfection

The BV2 microglial cell line was maintained in the culture medium containing DMEM (#10-013-CVR, Corning, New York, NY, USA) supplemented with 10% fetal bovine serum (#10270, Life Technologies, Carlsbad, CA, USA) and 1% Penicilin/Streptomicin (#30-001-CI, Corning, New York, NY, USA). For immunocytochemistry and for electrophysiological recordings, the cells were seeded at low density on 13 mm glass coverslips (#0117530, Marienfeld, Lauda-Königshofen, Germany) and on 35 mm culture Petri dishes (#353001, Corning, New York, NY, USA), respectively. For siRNA transfection, BV2 cells were cultured at a density of 3 × 10^6^ cell/mL in 6 well plates (#353224, Corning, New York, NY, USA) and kept in the incubator at 5% CO_2_ and 37 °C for 24 h. The cells were transfected using the INTERFERin^®^ reagent for siRNA (#89129, Polyplus transfection^®^, New York, NY, USA) diluted in the culture medium without serum and Penicilin/Streptomicin. The small interfering RNA was provided by siTOOLs Biotech, Planegg, Germany as a siPOOL-10 Kit containing the silencing RNA for the mouse gene ID #16518 coding for Kcnj2 (potassium inwardly rectifying channel, subfamily J, member 2), siPOOL-10 Kit containing the silencing RNA for the mouse gene ID #16491 coding for Kcna3 (potassium voltage-gated channel, shaker-related subfamily, member 3) and the siPOOL scramble negative control (see the Tables S1 and S2 for the full siRNA sequence). As previously described with minor changes [55], 8 μL of INTERFERin^®^ were mixed in 100 μL of serum-free medium containing 50 nM of silencing or scramble siRNA, vortexed, and incubated for 10 min at room temperature. Then, the transfection mix was completed up to 1 mL with the culture medium added on the BV2 culture for 24 h and 48 h at 5% CO_2_ and 37 °C.

#### 4.2.2. Primary Microglial Culture

Primary microglia cultures were prepared from the lumbar spinal cord at 2 days after the surgery. Animals were subjected to cervical dislocation and the spinal column was dissected and flushed from the sacral region with sterile phosphate buffer saline (PBS) to obtain the spinal cord. The lumbar ipsilateral SC-DH was minced in serum-free DMEM using a sterile scalpel. To dissociate the tissue, in the medium was added 2 mg/mL of papain (P3125, Sigma) and it was left for 45 min at 37 °C, 5% CO_2_, in the incubator. Afterwards, the cells were mechanically dissociated and centrifuged at 1500 rpm for 5 min. The cell sediment was re-suspended in the culture medium and filtered using 70 µm cell filters (#352350, Corning, New York, NY, USA), as previously described [56]. The cells were seeded in 8 µm pore inserts (#662638, Greiner Bio-One, Kremsmünster, Austria) using the culture medium in a volume of 200 µL/insert and 600 µL/well, to test the migration rate 3 h after the incubation at 37 °C, 5% CO_2_.

### 4.3. Migration

#### 4.3.1. BV2 Cell Line Migration

After a culture of 24 h, the confluent monolayer of BV2 cells was scratched using a sterile 200 µL pipette tip to obtain a robust and repetitive migration channel (Figure 3A and Figure 4A), as previously described [57]. To remove the loosely attached cells, the culture was washed with PBS before and after making the scratch. Afterwards, for the control conditions, the cells were maintained in the culture medium and for the treated conditions the cells were kept in the culture medium supplemented with 10 µM of UK78282 (#U3885, Sigma, St. Louis, MO, USA) to block Kv1.3, 10 µM of S9947 (#1657, Axon Medchem, Groningen, Netherlands) to block Kv1.5 and 20 µM of ML133 (#SML0190, Sigma, St. Louis, MO, USA) to inhibit Kir2.1 potassium channels. In the experiments regarding cell transfection, for control conditions the cells were transfected with 50 nM of scrambled siRNA and for the treated conditions the cells were transfected with 50 nM of silencing siRNA for Kv1.3 and for Kir2.1 potassium channels. Images were taken at different time points: t0h (immediately after the scratch), t24h (24 h after the scratch), and t48h (48 h after the scratch). When using pharmacological blockers, pictures were acquired with a 10× objective using an inverted Olympus IX73 microscope (Olympus, Tokyo, Japan) connected with a Hamamatsu camera (Hamamatsu Photonics, Hamamatsu City, Japan) controlled by the CellSens Dimension software (Olympus, Tokyo, Japan). After cell transfection, pictures were acquired with a 10× objective using a Zeiss Axio Vert.A1 inverted microscope connected with a AxioCam MRc5 Digital Camera controlled by the AxioVision SE64 Rel. 4.9.1 software (Zeiss, Jena, Germany). Subsequently, the pictures were saved and processed with the Fiji program [58]. The migration was assessed as a ratio of the cell free area, with a reduced area due to cell migration and proliferation, and a high remaining area due to the lack of migration. The cell free area of the scratch was analyzed at t24h and at t48h after the wound and the condition with the channel inhibitor or after transfection at t24h and at t48h were compared with the normalized control condition at t24h. Each experiment was repeated from at least three different cultures and the *n* values represent the number of pictures analyzed per condition.

#### 4.3.2. Primary Microglial Migration

Primary cultures of microglia were obtained from the CX3CR1-eGFP transgenic mice. The same concentrations of inhibitors were used in both the inserts and in the 24 well culture plate (#353047, Corning, New York, NY, USA) to assess the rate of migration. The cultures were incubated for 3 h, at 37 °C, 5% CO_2_, allowing the cells to migrate through the 8 µm pores. Afterwards, the inserts were fixed with 4% paraformaldehyde (PFA) (#252549; Sigma, St. Louis, MO, USA) for 5 min, stained with 2 µg/mL of Hoechst (#33342, Sigma, St. Louis, MO, USA) in PBS for 20 min in the dark and pictures were taken using an IX73 Olympus microscope and CellSens software. The images were processed with the Fiji software, all the experiments were replicated from at least 3 different animals in sham or SNI conditions. The migration rate was analyzed by counting the Hoechst-stained nuclei from the CX3CR1-eGFP microglial cells that migrated through the insert and the data are represented as normalized values. The *n* values represent the number of pictures analyzed for each condition.

### 4.4. Electrophysiology

Patch clamp recordings were made in whole-cell configuration. Borosilicate glass capillaries were used to produce micropipettes (Harvard Apparatus, Cambridge, MA, USA), with 1.5 mm OD, 0.86 mm ID, pulled with a vertical micropipette puller (Pull-100, World Precision Instruments, Sarasota, FL, USA), heat polished with a microforge to a resistance of 3–5 MΩ and filled with an intracellular solution consisting of (in mM): NaCl 5, KCl 130, MgCl_2_ 2, CaCl_2_ 1, HEPES 10, EGTA 10 (pH adjusted to 7.3 with KOH). The extracellular solution (applied in the bath) contains the following (in mM): NaCl 140, KCl 4, MgCl_2_ 1, CaCl_2_ 2, HEPES 10, NaOH 4.54, and glucose 7.4 (pH adjusted to 7.4). The cells were visualized under a Nikon Eclipse TE300 inverted microscope (Nikon, Tokyo, Japan). To reach the whole cell configuration, the micropipette was handled with a micromanipulator (Burleigh PCS5000 series). Currents were recorded at room temperature (22–25 °C), with an WPC-100 amplifier connected to a DigiData 1322A acquisition system (Molecular Devices, San Jose, CA, USA), with a sampling rate of 10 kHz, from a holding potential of −60 mV. The specific blockers were used at a concentration ten times less than for the migration test due to the high sensitivity of the electrophysiological recordings. Therefore, 1 µM of UK78282 for Kv1.3, 1 µM of S9947 for Kv1.5 and 2 µM of ML133 for Kir2.1 potassium channels were applied with the bath solution at a flow rate of 1–2 mL/min and the recordings were made before and after 3 min of acute application of the inhibitor. All pharmacological inhibitors used in this study are synthetic and present a high affinity and efficacy of blocking potassium channels, having an IC50 that ranges from approximately 200 nm in the case of UK78282 [50], 0.7 µM for S9947 [59] and 1.8 μM for ML133 [49].

### 4.5. Immunocytochemistry

BV2 cells were cultured on coverslips and then fixed in a 4% PFA solution in PBS for 20 min and incubated for 1 h in a blocking solution with 0.3% Triton X-100 and 3% bovine serum albumin (BSA) in PBS. The primary antibodies were added and incubated overnight at 4 °C, then the cells were washed with PBS, incubated for 1 h with the secondary antibody and mounted with Prolong Gold antifade with DAPI (#P10144, Life Technologies, Carlsbad, CA, USA). The potassium channels were visualized with primary antibodies anti-Kv1.3, anti-Kv1.5, anti-Kir2.1 (#APC-002, #APC-004, #APC-0026 Alomone, Jerusalem, Israel), used in a dilution of 1:300 and the secondary antibody was goat anti-rabbit Alexa Fluor 568 (1:1500, #A11011, Life Technologies, Carlsbad, CA, USA). The pictures were acquired using an inverted Olympus IX73 fluorescence microscope, processed with the CellSens and the Fiji software.

### 4.6. Analysis

pClamp 8.1 software (Molecular Devices) was used for the acquisition and analysis of patch clamp currents. Prism 5 software (GraphPad, San Diego, CA, USA) and Origin 8.5 software (OriginLab, Northampton, MA, USA) were used for the statistical analysis. For the migration experiments, the pictures were processed with the CellSens software and the cells were counted depending on the DAPI and GFP signals using Fiji, by an investigator blinded to the condition. The distribution was tested using the D’Agostino-Pearson omnibus normality test followed by an unpaired parametric t-test if the values passed the normality test, in the case of BV2 migration, or followed by a nonparametric Mann–Whitney statistical test if the values did not pass the normality test, in the case of the primary microglial migration. Values are given as Mean ± SD, *: *p* < 0.05, **: *p* < 0.01, ***: *p* < 0.001.

## 5. Conclusions

This study indicates the presence of the Kv1.3, Kv1.5 and Kir2.1 potassium channels at the plasma membrane of the BV2 microglial cell line using immunocytochemistry and electrophysiological recordings. In the scratch wound healing assay, only the Kv1.3 and Kir2.1 channels are implicated in BV2 microglial migration. After the SNI surgery, spinal microglial cells migrate less than in sham conditions, and their migration is reduced following the inhibition of the investigated potassium channels. However, in our hands, blocking Kv1.5 did not impact the migration of microglia in culture after SNI. Due to the importance of microglia responses to acute or chronic CNS diseases, understanding and modulating potassium channels involved in microglia responses to injury could provide potential drug targets on the long run.

## Figures and Tables

**Table 1:** Complete statistical results of primary microglial migration.

Sham	1 ± 0.4923, *n* = 70			
SNI	0.113 ± 0.13, *n* = 61, *p* < 0.001			
		K1.3 (UK78282)	Kv1.5 (S9947)	Kir2.1 (ML133)
Sham	Control	1 ± 0.4188, *n* = 21	1 ± 0.6214, *n* = 28	1 ± 0.3736, *n* = 21
	Inhibitor	0.425 ± 0.294, *n* = 21, *p* < 0.001	0.271 ± 0.2633, *n* = 28, *p* < 0.001	0.075 ± 0.06508, *n* = 21, *p* < 0.001
SNI	Control	1 ± 1.141, *n* = 21	1 ± 1.147, *n* = 21	1 ± 0.9143, *n* = 21
	Inhibitor	0.170 ± 0.2331, *n* = 21, *p* < 0.001	1.065 ± 1.415, *n* = 20, *p* > 0.05	0.447 ± 0.5559, *n* = 21, *p* < 0.05

## Data Availability

All data can be obtained upon request.

## Supplementary Materials

The following are available online at https://www.mdpi.com/1422-0067/22/4/2081/s1, Table S1: siPOOL sequences containing the silencing RNA for the mouse gene ID #16518 coding for Kcnj2 (potassium inwardly-rectifying channel, subfamily J, member 2), Table S2: siPOOL sequences containing the silencing RNA for the mouse gene ID #16491 coding for Kcna3 (potassium voltage-gated channel, shaker-related subfamily, member 3).

## Abbreviations

CNS	central nervous system
ROS	reactive oxygen species
NO	nitric oxide
LPS	lipopolysaccharide
SC-DH	spinal cord dorsal horn
SNI	spared nerve injury
I/V	intensity/voltage
siRNA	small interfering RNA
PBS	phosphate buffer saline
PFA	paraformaldehyde
BSA	bovine serum albumin
