# Supplementary material for: Potassium Channels Kv1.3 and Kir2.1 But Not Kv1.5 Contribute to BV2 Cell Line and Primary Microglial Migration
Source: Int J Mol Sci. 2021 Feb 19;22(4):2081. doi: 10.3390/ijms22042081 (PMC7923211; doi:10.3390/ijms22042081)
Supplement: Supplementary file 1 [file ijms-22-02081-s001.pdf]

Tables

TABLE S1 siPOOL sequences containing the silencing RNA for the mouse gene ID #16518 coding for Kcnj2 (potassium inwardly-rectifying channel, subfamily J, member 2)

Row	siPOOL	Target NCBI Gene ID	Target symbol	Target species	siRNA number	Sense seq	Antisense seq
1	Kcnj2-m	16518	Kcnj2	mouse	1	GCCGCTTTGTGAAGAAAGA	TCTTTCTTCACAAAGCGGC
2	Kcnj2-m	16518	Kcnj2	mouse	2	GCACAGCTTCTCAAATCTA	TAGATTTGAGAAGCTGTGC
3	Kcnj2-m	16518	Kcnj2	mouse	3	GGGAGTATATCCCTTTGGA	TCCAAAGGGATATACTCCC
4	Kcnj2-m	16518	Kcnj2	mouse	4	GAGGCGACTGCCATGACAA	TTGTCATGGCAGTCGCCTC
5	Kcnj2-m	16518	Kcnj2	mouse	5	GCATAGATCTCCACAACCA	TGGTTGTGGAGATCTATGC
6	Kcnj2-m	16518	Kcnj2	mouse	6	GAGAGTGGGTAACCTTCGA	TCGAAGGTTACCCACTCTC
7	Kcnj2-m	16518	Kcnj2	mouse	7	GTGACAGACGAGTGCCCAA	TTGGGCACTCGTCTGTCAC
8	Kcnj2-m	16518	Kcnj2	mouse	8	GACTATTCAAGATTCCATA	TATGGAATCTTGAATAGTC
9	Kcnj2-m	16518	Kcnj2	mouse	9	GTAAAGTAATCAAACCAAA	TTTGGTTTGATTACTTTAC
10	Kcnj2-m	16518	Kcnj2	mouse	10	GCACTGTAGTCATTTCTAA	TTAGAAATGACTACAGTGC
11	Kcnj2-m	16518	Kcnj2	mouse	11	GCAATGTTCAGTTTATCAA	TTGATAAACTGAACATTGC
12	Kcnj2-m	16518	Kcnj2	mouse	12	GGTTATTTCTCTCTCTGTA	TACAGAGAGAGAAATAACC
13	Kcnj2-m	16518	Kcnj2	mouse	13	CCAGAGACTTAGCAGAGAA	TTCTCTGCTAAGTCTCTGG
14	Kcnj2-m	16518	Kcnj2	mouse	14	GGTATTCCAGTCAATCGTA	TACGATTGACTGGAATACC
15	Kcnj2-m	16518	Kcnj2	mouse	15	GCCAGTAATTTGTTTGCTA	TAGCAAACAAATTACTGGC
16	Kcnj2-m	16518	Kcnj2	mouse	16	GACTTGAGTAAGCAGGACA	TGTCCTGCTTACTCAAGTC
17	Kcnj2-m	16518	Kcnj2	mouse	17	GTGTGAGAACCAACCGCTA	TAGCGGTTGGTTCTCACAC
18	Kcnj2-m	16518	Kcnj2	mouse	18	CTTTGAAATTGTTGTCATA	TATGACAACAATTTCAAAG
19	Kcnj2-m	16518	Kcnj2	mouse	19	CACTATCGTTCACGAAATA	TATTTCGTGAACGATAGTG
20	Kcnj2-m	16518	Kcnj2	mouse	20	GCCGGTCTGTGGCTTGTGA	TCACAAGCCACAGACCGGC
21	Kcnj2-m	16518	Kcnj2	mouse	21	GCCCAGGCCCTTAAGGCGA	TCGCCTTAAGGGCCTGGGC
22	Kcnj2-m	16518	Kcnj2	mouse	22	CCATAAGACTTATGAAGTA	TACTTCATAAGTCTTATGG
23	Kcnj2-m	16518	Kcnj2	mouse	23	GAGTGATTTTAATGGCATA	TATGCCATTAAAATCACTC
24	Kcnj2-m	16518	Kcnj2	mouse	24	GAAGAGAAACACTACTATA	TATAGTAGTGTTTCTCTTC
25	Kcnj2-m	16518	Kcnj2	mouse	25	GCGAAGATGGCAAAGCCAA	TTGGCTTTGCCATCTTCGC
26	Kcnj2-m	16518	Kcnj2	mouse	26	GCAAAGCATGCGTGTCAGA	TCTGACACGCATGCTTTGC
27	Kcnj2-m	16518	Kcnj2	mouse	27	GGGTCACCGCTATGAGCCA	TGGCTCATAGCGGTGACCC
28	Kcnj2-m	16518	Kcnj2	mouse	28	GGGTCATTGTCTCTTGGAA	TTCCAAGAGACAATGACCC
29	Kcnj2-m	16518	Kcnj2	mouse	29	GGGACAGAGGTACCTGGCA	TGCCAGGTACCTCTGTCCC
30	Kcnj2-m	16518	Kcnj2	mouse	30	GCTAATAGGTTATCTCAAA	TTTGAGATAACCTATTAGC

TABLE S2 siPOOL sequences containing the silencing RNA for the mouse gene ID #16491 coding for Kcna3 (potassium voltage-gated channel, shaker-related subfamily, member 3)

Row	siPOOL	Target NCBI Gene ID	Target symbol	Target species	siRNA number	Sense seq	Antisense seq
1	Kcna3-m	16491	Kcna3	mouse	1	GCTTCGACGCCATCCTCTA	TAGAGGATGGCGTCGAAGC
2	Kcna3-m	16491	Kcna3	mouse	2	CTCATCAAGGAAAGTACAA	TTGTACTTTCCTTGATGAG
3	Kcna3-m	16491	Kcna3	mouse	3	CTCCGAAAAGCCCGGAGTA	TACTCCGGGCTTTTCGGAG
4	Kcna3-m	16491	Kcna3	mouse	4	GGTGGGCAGTAGTAACCAT	ATGGTTACTACTGCCCACC
5	Kcna3-m	16491	Kcna3	mouse	5	CTGGCCATCCTGAGAGTCA	TGACTCTCAGGATGGCCAG
6	Kcna3-m	16491	Kcna3	mouse	6	GTGGAGACCTTGTGCATCA	TGATGCACAAGGTCTCCAC
7	Kcna3-m	16491	Kcna3	mouse	7	CGGTCAACGTGCCCATCGA	TCGATGGGCACGTTGACCG
8	Kcna3-m	16491	Kcna3	mouse	8	CTGGCTGAACGACAAGGTA	TACCTTGTCGTTCAGCCAG
9	Kcna3-m	16491	Kcna3	mouse	9	CCATGACAACTGTTGGTTA	TAACCAACAGTTGTCATGG
10	Kcna3-m	16491	Kcna3	mouse	10	CCGAGACACTGCTGGGCGA	TCGCCCAGCAGTGTCTCGG
11	Kcna3-m	16491	Kcna3	mouse	11	GCTTGGAGACGCTTCCCGA	TCGGGAAGCGTCTCCAAGC
12	Kcna3-m	16491	Kcna3	mouse	12	GGTGAGGAGGCCATGGAAA	TTTCCATGGCCTCCTCACC
13	Kcna3-m	16491	Kcna3	mouse	13	GCTTTTACCAGCTGGGTGA	TCACCCAGCTGGTAAAAGC
14	Kcna3-m	16491	Kcna3	mouse	14	CCAAGCGGCGCATGCGGTA	TACCGCATGCGCCGCTTGG
15	Kcna3-m	16491	Kcna3	mouse	15	GCATTGCCAGTTCCTGTGA	TCACAGGAACTGGCAATGC
16	Kcna3-m	16491	Kcna3	mouse	16	GCCATCGCAGGTGTCTTGA	TCAAGACACCTGCGATGGC
17	Kcna3-m	16491	Kcna3	mouse	17	GCCACTTGCACCACGAACA	TGTTCGTGGTGCAAGTGGC
18	Kcna3-m	16491	Kcna3	mouse	18	CCCGAGTTTCGCGATGAGA	TCTCATCGCGAAACTCGGG
19	Kcna3-m	16491	Kcna3	mouse	19	CACAGCGCCTTCCCGCAGA	TCTGCGGGAAGGCGCTGTG
20	Kcna3-m	16491	Kcna3	mouse	20	GACCCTTCTTCGGGTTTTA	TAAAACCCGAAGAAGGGTC
21	Kcna3-m	16491	Kcna3	mouse	21	GTGTTTGAGGCTGCCAACA	TGTTGGCAGCCTCAAACAC
22	Kcna3-m	16491	Kcna3	mouse	22	GGTGATATGCACCCAGTGA	TCACTGGGTGCATATCACC
23	Kcna3-m	16491	Kcna3	mouse	23	GAGAGTCATCCGCCTAGTA	TACTAGGCGGATGACTCTC
24	Kcna3-m	16491	Kcna3	mouse	24	CGTCGCAGGACGTGTTTGA	TCAAACACGTCCTGCGACG
25	Kcna3-m	16491	Kcna3	mouse	25	CCATGGAAAAGTTCCGTGA	TCACGGAACTTTTCCATGG
26	Kcna3-m	16491	Kcna3	mouse	26	CTGCAGATCCTAGGACAGA	TCTGTCCTAGGATCTGCAG
27	Kcna3-m	16491	Kcna3	mouse	27	CGGAGTAACTCCACTCTGA	TCAGAGTGGAGTTACTCCG
28	Kcna3-m	16491	Kcna3	mouse	28	CTGAGTAAGTCGGAGTATA	TATACTCCGACTTACTCAG
29	Kcna3-m	16491	Kcna3	mouse	29	GATATTCACTGATGTCTAA	TTAGACATCAGTGAATATC
30	Kcna3-m	16491	Kcna3	mouse	30	CCAGAAATATCATGAACTT	AAGTTCATGATATTTCTGG
